# A viral metagenomic survey identifies known and novel mammalian viruses in bats from Saudi Arabia

**DOI:** 10.1371/journal.pone.0214227

**Published:** 2019-04-10

**Authors:** Nischay Mishra, Shamsudeen F. Fagbo, Abdulaziz N. Alagaili, Adam Nitido, Simon H. Williams, James Ng, Bohyun Lee, Abdulkareem Durosinlorun, Joel A. Garcia, Komal Jain, Vishal Kapoor, Jonathan H. Epstein, Thomas Briese, Ziad A. Memish, Kevin J. Olival, W. Ian Lipkin

**Affiliations:** 1 Center for Infection and Immunity, Mailman School of Public Health, Columbia University, New York, New York, United States of America; 2 One Health Unit, Executive Directorate for Surveillance and Response, National Center for Disease Prevention and Control, Riyadh, Saudi Arabia; 3 KSU Mammals Research Chair, Department of Zoology, College of Science, King Saud University, Riyadh, Saudi Arabia; 4 Federal Ministry of Agriculture and Rural Development, Kaduna, Nigeria; 5 EcoHealth Alliance, New York, New York, United States of America; 6 The College of Medicine, Al faisal University & Prince Mohammed Bin Abdulaziz Hospital, Ministry of Health, Riyadh, Kingdom of Saudi Arabia; University of Pretoria, SOUTH AFRICA

## Abstract

Bats are implicated as natural reservoirs for a wide range of zoonotic viruses including SARS and MERS coronaviruses, Ebola, Marburg, Nipah, Hendra, Rabies and other lyssaviruses. Accordingly, many One Health surveillance and viral discovery programs have focused on bats. In this report we present viral metagenomic data from bats collected in the Kingdom of Saudi Arabia [KSA]. Unbiased high throughput sequencing of fecal samples from 72 bat individuals comprising four species; lesser mouse-tailed bat (*Rhinopoma hardwickii)*, Egyptian tomb bat *(Taphozous perforatus)*, straw-colored fruit bat *(Eidolon helvum)*, *and* Egyptian fruit bat *(Rousettus aegyptiacus*) revealed molecular evidence of a diverse set of viral families: *Picornaviridae* (hepatovirus, teschovirus, parechovirus), *Reoviridae* (rotavirus), *Polyomaviridae* (polyomavirus), *Papillomaviridae* (papillomavirus), *Astroviridae* (astrovirus), *Caliciviridae* (sapovirus), *Coronaviridae* (coronavirus), *Adenoviridae* (adenovirus), *Paramyxoviridae* (paramyxovirus), and unassigned mononegavirales (chuvirus). Additionally, we discovered a bastro-like virus (Middle East Hepe-Astrovirus), with a genomic organization similar to *Hepeviridae*. However, since it shared homology with *Hepeviridae* and *Astroviridae* at ORF1 and in ORF2, respectively, the newly discovered Hepe-Astrovirus may represent a phylogenetic bridge between *Hepeviridae* and *Astroviridae*.

## Introduction

Bats (order Chiroptera) are the second most diverse mammalian order after rodents, comprising more than 1400 species that represent approximately 20% of all mammalian species [1]. Bats are beneficial in arthropod suppression, seed dispersal, and pollination; and serve as a bushmeat food source for some human populations [2, 3]. Bats are also natural reservoirs for a wide range of emerging viruses that may cause infectious diseases in humans, livestock, domestic animals and wildlife [4], and as a group bats also harbor a higher proportion of zoonotic viruses than other mammals [5]. As with most groups of mammals, bats are predicted to have a large diversity of viruses, which are currently undescribed [5, 6]. The development of more powerful high throughput sequencing (HTS) technology and bioinformatic tools now allow us to better characterize this viral diversity, including analysis of samples from geographic regions that have received little wildlife surveillance attention to date. Recently several bat virome studies have been published using high-throughput viral metagenomic approaches, revealing a wide range of viruses and expanding our knowledge of viral diversity. A coronavirus similar to Middle East respiratory syndrome coronavirus (MERS-CoV), adenoviruses, herpesviruses and circoviruses were reported in *Neoromicia capensis* bats from South Africa. Yinda et. al reported diverse rotaviruses, bastroviruses and sapoviruses using HTS analysis of *Eidolon helvum* and *Epomophorus gambianus* bats from Cameroon [7–10].

During the course of an investigation into potential animal reservoirs for the MERS-CoV in the Kingdom of Saudi Arabia [KSA], we trapped and collected fecal specimens from individuals representing four bat species (*R*. *hardwickii*, *T*. *perforatus*, *E*. *helvum and R*. *aegyptiacus*) located in close proximity to the human index case in the town of Bisha, KSA [11]. High throughput viral metagenomic analyses of fecal samples revealed the presence of several known and novel bat RNA and DNA viruses in all four species. We recovered whole genomes of hepatovirus, rotavirus, polyomavirus, and partial genomes of papillomavirus, astrovirus, sapovirus, teschovirus, parechovirus, coronavirus, adenovirus, paramyxovirus, and chuvirus. In both *R*. *hardwickii* and *E*. *helvum*, we also found evidence of a virus that, like the recently reported bastrovirus, has elements of both hepeviruses and astroviruses, but differs in genomic organization. The Middle East Hepe-Astrovirus (ME Hepe-Astrovirus) has a genomic organization similar to *Hepeviridae*; however, it shares homology in certain regions of the genome to members from two viral families, *Hepeviridae* and *Astroviridae*.

## Material and methods

### Sample collection and library preparation

In October 2012, fecal samples from 72 bat individuals were collected from 3 different sites in or around the town of Bisha, KSA [11]. Bat capture, morphological measurements and fecal sample collection were conducted as part of an outbreak investigation and surveillance effort with permission from and in coordination with the KSA Ministry of Health (Zoonotic Disease section). No methods for anesthesia, euthanasia, or any kind of animal sacrifice were involved in bat capturing and fecal sample collection. All bats were released after sampling. There was no local IACUC available in KSA at the time of this investigation; however, all bat sampling and handling were carried out in accordance with protocols from previously IACUC approved EcoHealth Alliance studies [12, 13]. All sampling was performed using minimally invasive (non-lethal) techniques detailed in a published protocol [14]. Briefly, individual bats were captured in either mist nets or harp traps located within ten meters of roosting sites or at foraging sites, and held in individual cloth bags until sampling. Bats were restrained manually while blood, urine, oral swabs, and rectal swabs/fecal samples were collected. Microbats (*R*. *hardwickii and T*. *perforatus)* were restrained between the handler’s thumb and palm of the non-preferred hand with wing extended with 90° angle between bat’s forearm and upper arm, the skin was wiped with alcohol and venous blood from brachial or propatagial (cephalic) vein was collected using a 25 or 27-gauge needle. Larger bats (*R*. *aegyptiacus* and *E*. *helvum*) were restrained by one researcher and second collected venous blood from propatagial or saphenous (inter-fermoral) vein [15].

External morphological measurements were taken and wing biopsy specimens were also taken to confirm species identification by DNA analysis. All bats were released at the site of capture immediately after sampling. Rectal swab samples and fecal pellet samples were stored in viral transport medium. Rectal swab and fecal pellet suspensions (2% weight/volume in 1X phosphate buffered saline) were filtered through a 0.45 μm spin column (Millipore-Sigma, St. Louis, MO) by centrifugation for 10 minutes (~6000 rpm) to remove host cells and other microorganisms. Filtered suspensions were then treated with DNAse and RNAse to enrich the viral sequences by destroying exogenous host, bacterial, and fungal nucleic acids without affecting nucleic acid protected inside the viral capsid or envelope [16]. Total nucleic acids were then extracted using EasyMag automated extraction platform (bioMérieux, Boxtel, The Netherlands) [17]. Superscript III and random hexamer primers were used to generate first stranded cDNA (Life Technologies, Carlsbad, CA, USA). Second-stranded cDNA synthesis for HTS was carried out by random primer extension with Klenow enzyme (New England Biolabs, Ipswich, MA, USA). Double stranded DNA preparations were sheared (E210 sonicator; Covaris, Woburn, MA, USA) for an average fragment size of 200 bp and added to Agencourt AMPure XP beads (Beckman Coulter, Brea, CA, USA) for purification, and libraries were prepared with the Hyper Prep kit (KAPA Biosystems, Wilmington, MA, USA). Sequencing was performed using a read length of 100-nt, followed by an independent read of the 6-nt barcode. Unbiased high-throughput sequencing was done on the HiSeq 2500 platform (Illumina, San Diego, CA, USA).

### Data analysis and bioinformatics pipeline

A total of 304 million reads were generated by Illumina HiSeq 2500, which were de-multiplexed using bcl2fastq software v 1.8.4 into individual samples for further analysis. The de-multiplexed HTS data was submitted to SRA NCBI (accession id: SRP158542). Reads were preprocessed to exclude host sequences and quality filtered prior to de novo assembly. Low quality and poor complexity reads were filtered using PRINSEQ (v 0.20.2) [18] and remaining reads were mapped to a host database using Bowtie2 mapper 2.0.6 (http://bowtie-bio.sourceforge.net). A host database was built using four bat genomic and ribosomal sequences (*Myotis lucifugus*, *Myotis davidii*, *Pteropus vampyrus*, *and Pteropus alecto*). The resulting reads were de novo assembled using MIRA (v4.0)[19] assembler and homology search was performed on NCBI GenBank database. Assembled contiguous sequences (contigs) and unique singletons from assembly were subjected to NCBI BLASTn, and unannotated sequences were further searched on non-redundant (*nr*) database using NCBI BLASTx. Based on BLASTn and BLASTx analysis, viral sequences matching with Illumina reads and contigs were downloaded from NCBI and used for mapping to recover partial or complete genomes.

### Phylogenetic analyses

Nucleotide and protein alignments were performed using MEGA 5.0 and MEGA 7.0 [20, 21]. Full genome assembly and annotation were performed using Geneious 10.1.2 [22]. Phylogenetic trees were constructed using MEGA 5.0/MEGA 7.0. The ME Hepe-Astrovirus phylogenetic tree was generated using BEAST2 Bayesian phylogenetic software [23] with WAG substitution model [24]. A tangelogram [25] was constructed in R using the dendextend package [26]. Recombination analyses were carried out using Simplot software [27].

### Accession number(s)

Genbank accession numbers for reported viruses are summarized in Table 1.

**Table 1:** Sequences, sources, and GenBank accession numbers.

Sample ID	Bat species	Virus	Gene region (partial or complete)	Fragment size [nt]	Protein [aa]	Accession number
KSA212	*Rhinopoma hardwickii*	Bat hepatovirus	5’ UTR (partial)	356	NA	MH367854
KSA212	*Rhinopoma hardwickii*	Bat hepatovirus	VP2 (partial)	261	87	MH367861
KSA215	*Rhinopoma hardwickii*	Bat hepatovirus	5’ UTR (partial)	356	NA	MH367852
KSA215	*Rhinopoma hardwickii*	Bat hepatovirus	VP2 (partial)	261	87	MH367858
KSA215	*Rhinopoma hardwickii*	Bat hepatovirus	Complete genome	7473	2228	KX420952
KSA218	*Rhinopoma hardwickii*	Paramyxovirus	Nucleocapsid protein (partial)	713	237	MH424434
KSA225	*Rhinopoma hardwickii*	Bat hepatovirus	5’ UTR (partial)	356	NA	MH367855
KSA225	*Rhinopoma hardwickii*	Bat hepatovirus	VP2 (partial)	261	87	MH367860
KSA226	*Rhinopoma hardwickii*	Bat hepatovirus	5’ UTR (partial)	356	NA	MH367857
KSA226	*Rhinopoma hardwickii*	Bat hepatovirus	VP2 (partial)	261	87	MH367859
KSA229	*Rhinopoma hardwickii*	Bat hepatovirus	5’ UTR (partial)	356	NA	MH367856
KSA230	*Rhinopoma hardwickii*	Bat hepatovirus	VP2 (partial)	261	87	MH367863
KSA236	*Rhinopoma Hardwickii*	Rotavirus	VP1 (partial)	658	219	MH673597
KSA238	*Rhinopoma hardwickii*	Bat hepatovirus	5’ UTR (partial)	356	NA	MH367853
KSA238	*Rhinopoma hardwickii*	Bat hepatovirus	VP2 (partial)	261	87	MH367862
KSA239	*Rhinopoma hardwickii*	ME Hepe-Astro virus	Complete genome	5839	2297	KX272763
KSA282	*Eidolon helvum*	Bat Coronavirus	RdRp (partial)	400	133	MH396479
KSA285	*Eidolon helvum*	Bat Coronavirus	RdRp (partial)	399	133	MH396478
KSA299	*Eidolon helvum*	Bat Coronavirus	RdRp (partial)	389	129	MH396477
KSA302	*Taphozous perforatus*	Bat Coronavirus	RdRp (partial)	398	132	MH396476
KSA402	*Eidolon helvum*	Rotavirus	VP1 (partial)	611	203	MH673598
KSA402	*Eidolon helvum*	Rotavirus	VP1 (complete)	3429	1089	KX420939
KSA402	*Eidolon helvum*	Rotavirus	VP2 (complete)	2846	904	KX420940
KSA402	*Eidolon helvum*	Rotavirus	VP3 (complete)	2716	836	KX420941
KSA402	*Eidolon helvum*	Rotavirus	VP4 (complete)	2455	777	KX420942
KSA402	*Eidolon helvum*	Rotavirus	NSP1 (complete)	2119	563	KX420943
KSA402	*Eidolon helvum*	Rotavirus	VP6 (complete)	1697	398	KX420944
KSA402	*Eidolon helvum*	Rotavirus	NSP2 (complete)	1455	318	KX420945
KSA402	*Eidolon helvum*	Rotavirus	VP7 (complete)	1300	327	KX420946
KSA402	*Eidolon helvum*	Rotavirus	NSP3 (complete)	1167	312	KX420947
KSA402	*Eidolon helvum*	Rotavirus	NSP5/6 (complete)	723	299	KX420948
KSA402	*Eidolon helvum*	Rotavirus	NSP4 (complete)	555	176	KX420949
KSA403	*Eidolon helvum*	Parechovirus	Polyprotein (partial)	1080	360	MH396474
KSA403	*Eidolon helvum*	Sapovirus	Nonstructural polyprotein and VP1 (partial)	896	298	MH396475
KSA403	*Eidolon helvum*	Bat polyomavirus	Complete genome	4696	1558	KX434762
KSA403	*Eidolon helvum*	Me Hepe-Astro virus	Complete genome	5745	2406	KX420950
KSA410	*Eidolon helvum*	Rotavirus	VP1 (partial)	657	219	MH673599
KSA410	*Eidolon helvum*	ME Hepe-Astro virus	partial genome	4911	1949	KX420951
KSA412	*Eidolon helvum*	Teschovirus	Polyprotein (partial)	3167	972	KX420938
KSA416	*Eidolon helvum*	Bat papillomavirus	Genome (complete coding regions)	6985	2328	KX434763
KSA419	*Eidolon helvum*	Bat astrovirus	RdRp (partial)	423	141	MH424430
KSA422	*Taphozous perforatus*	Bat astrovirus	RdRp (partial)	422	140	MH424431
KSA424	*Taphozous perforatus*	Bat papillomavirus	Genome (complete coding regions)	7180	2367	KX434764
KSA425	*Taphozous perforatus*	Bat mastadenovirus	Hexon (complete)	2700	900	KX434765
KSA428	*Taphozous perforatus*	Bat astrovirus	RdRp (partial)	422	140	MH424432
KSA428	*Taphozous perforatus*	Bat papillomavirus	Genome (partial)	3652	1215	KX434766
KSA431	*Taphozous perforatus*	Chuvirus	RdRp (partial)	762	254	MH396473
KSA433	*Taphozous perforatus*	Bat papillomavirus	Genome (complete coding regions)	7083	2321	KX434767
KSA434	*Taphozous perforatus*	Bat astrovirus	RdRp (partial)	422	140	MH424433
KSA435	*Taphozous perforatus*	Bat astrovirus	capsid (partial)	2564	854	MH424428
KSA435	*Taphozous perforatus*	Bat astrovirus	RdRp (partial)	422	140	MH424429
KSA435	*Taphozous perforatus*	Rotavirus	VP1 (partial)	616	205	MH673600
KSA624	*Eidolon helvum*	Rotavirus	VP1 (partial)	646	215	MH673601

## Results

### Virome analysis

We collected fecal samples from 72 individual bats representing four species from three bat families (*Pteropodidae*, *Rhinopomatidae*, and *Emballonuridae*) roosting in Bisha, KSA (Table 2). Bat species were identified using external morphological features [28] by trained bat biologists and confirmed via molecular testing by cytochrome B gene PCR amplification and sequencing [11, 29]. Total nucleic acid was extracted from fecal samples prior to library preparation and sequencing on the HiSeq 2500 system (Illumina, San Diego, CA).

**Table 2:** Description of bat specimens used for virome analyses.

Family	Species (n = no. of samples)	Common name	Diet	Sample type
Rhinopomatidae	Rhinopoma hardwickii (n = 29)	Lesser Mouse-tailed bat	Insectivorous	Feces/rectal swab
Emballonuridae	Taphozous perforatus (n = 17)	Egyptian tomb bat	Insectivorous	Feces/rectal swab
Pteropodidae	Eidolon helvum (n = 24)	Straw-colored fruit bat	Frugivorous	Feces/rectal swab
Pteropodidae	Rousettus aegyptiacus (n = 2)	Egyptian fruit bat	Frugivorous	Feces/rectal swab

**Fig 1:**
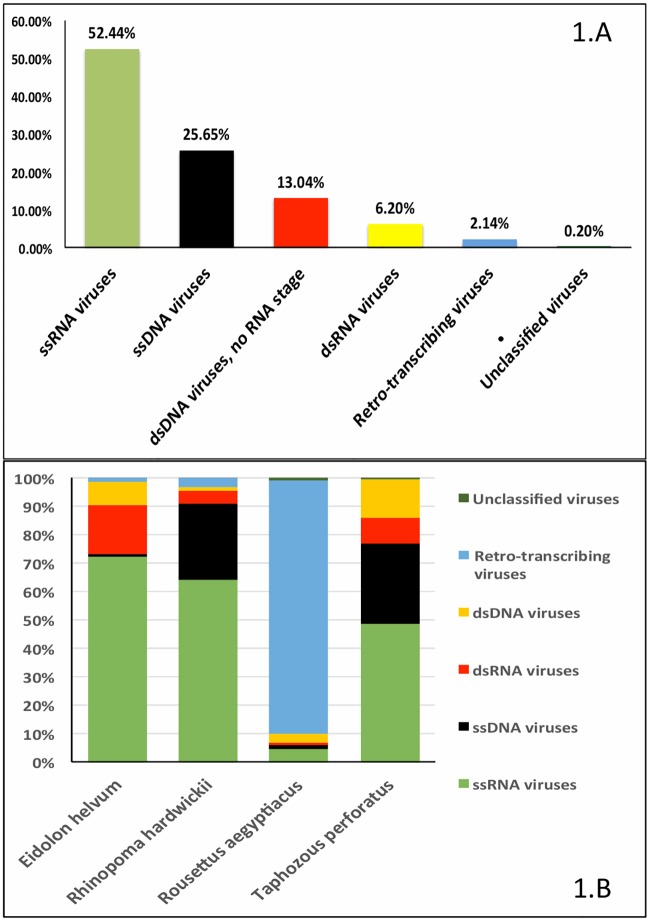
Overview of viruses detected through high throughput sequencing in the entire population (A) and by bat species (B).

The 72 libraries generated a total of 304 million single end 100 nt reads. Sequencing of the *E*. *helvum* (n = 24) pool yielded 113 million reads, *R*. *hardwickii* (n = 29) pool yielded 116 million reads, *T*. *perforates* (n = 17) pool yielded 68 million reads and *R*. *aegyptiacus* (n = 2) pool yielded 7 million reads. A total of 124 million reads (~40.7%) were identified as bat host sequences and 13.7 million (~4.5%) low quality and low complexity reads were removed by filtering using PRINSEQ (v 0.20.2)[18]. Of the remaining 167 million reads, 31 million were shared between fungal (~0.43 million, ~0.3%), bacterial (~29 million, ~17.3%), phage (~0.27 million, ~0.2%) and viral sequences (1.6 million, ~0.52%). The remaining ~130 million reads had similarity with genomic sequences of plants, insects, vertebrates and invertebrates, which largely can be explained by the dietary preference of each bat species. Viral results showed sequences matching with a wide range of known and novel viruses. Approximately 1.6 million (~0.52% of total reads) had nt or amino acid [aa] homology with the entries in the NCBI viral database [30] that included ssRNA viruses (~0.97 million reads, 52.4% of viral reads), ssDNA viruses (~0.47 million reads, 25.7%), dsRNA viruses (~0.11 million reads, 6.2%), dsDNA viruses (~0.24 million reads, 13.0%), retro-transcribing viruses (~0.04 million reads, 2.1%) and unclassified viruses (~0.004 million reads, 0.2%) (Fig 1). The virus population distribution by Baltimore classification [31] was different amongst the four bat species. Whereas results for *R*. *hardwickii*, *T*. *perforatus*, and *E*. *helvum* represent 17 or more bats per species, results for *R*. *aegyptiacus* reflect analysis of only two bats. Reads and contigs related to ssRNA viruses were abundant in *R*. *hardwickii*, *T*. *perforatus and E*. *helvum* bats (50–72%). *R*. *aegyptiacus* was dominated by retro-transcribing viruses (~88%). Reads and contigs related to ssRNA viruses were found in higher number in *E*. *helvum* and *R*. *hardwickii* (Fig 1). Sequences related to ssDNA viruses were found in higher number in *T*. *perforatus* and *R*. *hardwickii* (Fig 1). More than 50% of the ssRNA viral reads represented insect-borne picornaviruses that are presumably the result of an insectivorous diet. Similarly, greater than 70% of the ssDNA viral reads represented insect-borne densoviruses and parvoviruses. Based on BLASTn/BLASTx analyses, representations of viral reads per bat species are described in S1 Fig. Sequencing data also indicated the presence of bacteriophages, animal and plant viruses. Database queries (BLASTn/BLASTx) identified contigs with low homology to known RNA and DNA viruses of vertebrates.

There were differences in viral distribution and relative presence between all four species (S1 Fig). Single stranded RNA viruses were most abundant in *R*. *hardwickii* and least abundant in *R*. *aegyptiacus*. Insect-borne ssRNA viruses from family *Dicistroviridae* were most abundant in *R*. *hardwickii* (insectivorous bats). Most ssRNA virus reads found in *E*. *helvum* and *R*. *aegyptiacus* samples were from the *Picornaviridae* family. Unclassified ssRNA negative-stranded viruses were most abundant in *T*. *perforatus*. Fecal material from *R*. *hardwickii* contained the most diverse population of ssRNA viruses (S1 Fig). Single stranded DNA viruses showed least diversity in all four-bat species. For the two frugivorous bats [family Pteropodidae] sampled, unclassified parvoviruses were most common (68%) in *E*. *helvum* bats while unclassified circoviruses and ssDNA viruses were most common (74%) in *R*. *aegyptiacus*. Insect-borne ssDNA densoviruses were most common in *R*. *hardwickii* (insectivorous bats). Unclassified insect-borne ssDNA parvoviruses were most common in *T*. *perforatus* (insectivorous bats) (S1 Fig). Reads and contigs from *Adenoviridae* family were most common in *T*. *perforatus bats*; sequences sharing identity with family *Polyomaviridae* were most common in *E*. *helvum* and *R*. *aegyptiacus*, and baculoviruses sequences were most common in *R*. *hardwickii* (S1 Fig). Sequences matching an insect-borne Drosophila A virus dsRNA virus were most abundant in *R*. *hardwickii* (insectivorous bats). Rotavirus reads were most abundant (~80%) in *E*. *helvum* (S1 Fig).

Single stranded RNA and dsRNA virus diversity was more pronounced in *R*. *hardwickii* than in other bat species. *E*. *helvum* and *R*. *aegyptiacus* bat species showed >50% ssRNA viral reads representing the order *Picornavirales*. *T*. *perforatus* and *E*. *helvum* had more unclassified negative stranded ssRNA viral reads than either *R*. *aegyptiacus* or *R*. *hardwicki*. *R*. *aegyptiacus* and *R*. *hardwickii* had more papillomavirus reads than *E*. *helvum* and *T*. *perforatus*. In further analysis of sequence contigs with homology to viruses of vertebrate origin we found that some viruses appeared to be restricted to specific bat species (e.g. hepatovirus in *R*. *hardwickii*, sapovirus in *E*. *helvum*, adenovirus in *T*. *perforatus*). Descriptions of sequences and Genbank acc. numbers are summarized in Table 1.

### Single stranded RNA Viruses

**Fig 2:**
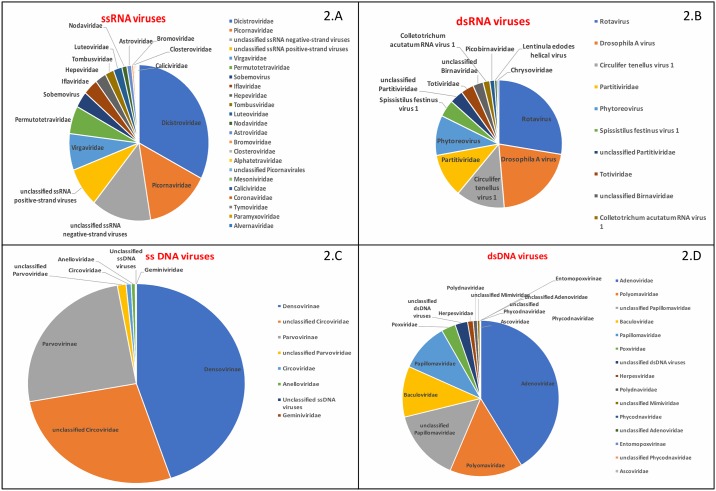
Comparative abundance of sequences representing viral taxa within the larger categories of: Single stranded RNA viruses (A), Double stranded RNA viruses (B), Single stranded DNA viruses (C), and Double stranded DNA viruses (D).

Approximately 0.97 million (52.4%) reads matched to ssRNA viruses (Fig 2). Of these reads, approximately 50% represented *Picornavirales* (~33% to family *Dicsitroviridae* and ~15% *Picornaviridae*); ~18% reads represented plant viral families (*Virgaviridae*, *Permutotetraviridae*, *Sobemovirus*). Of the total ssRNA virus reads, 22% reads matched with unclassified viruses (3.2% with positive sense ssRNA and 13.8% with negative sense ssRNA viruses). The remaining 7% ssRNA viral reads revealed the presence of bastro-like virus (ME Hepe-Astrovirus), hepatoviruses, astroviruses, hepeviruses and coronaviruses.

#### Middle East Bat Hepe-Astroviruses

Hepeviruses are non-enveloped, single-stranded, positive sense RNA viruses with ~7.2 kb genome, containing a 5’ non-coding region (NCR), 3 open reading frames (ORFs), and a short 3’ NCR terminated by a poly-A tail [32]. Astroviruses are also non-enveloped, positive-sense ssRNA viruses (6–8 kb genome) with 5’ and 3’ NCRs and three ORFs [33]. HTS data revealed sequences found in three bat fecal samples (two from *E*. *helvum* and one from *R*. *hardwickii*) that shared identity with recently discovered bastroviruses in human feces from the Netherlands and *E*. *helvum* feces from Cameroon [9, 34]. Bastroviruses are tentatively classified as astroviruses. The putative structural protein of Bastrovirus shared homology to the capsid protein of *Astroviridae* and the putative non-structural protein was homologous to the non-structural protein encoded by members of the *Hepeviridae* family [34].

**Fig 3:**
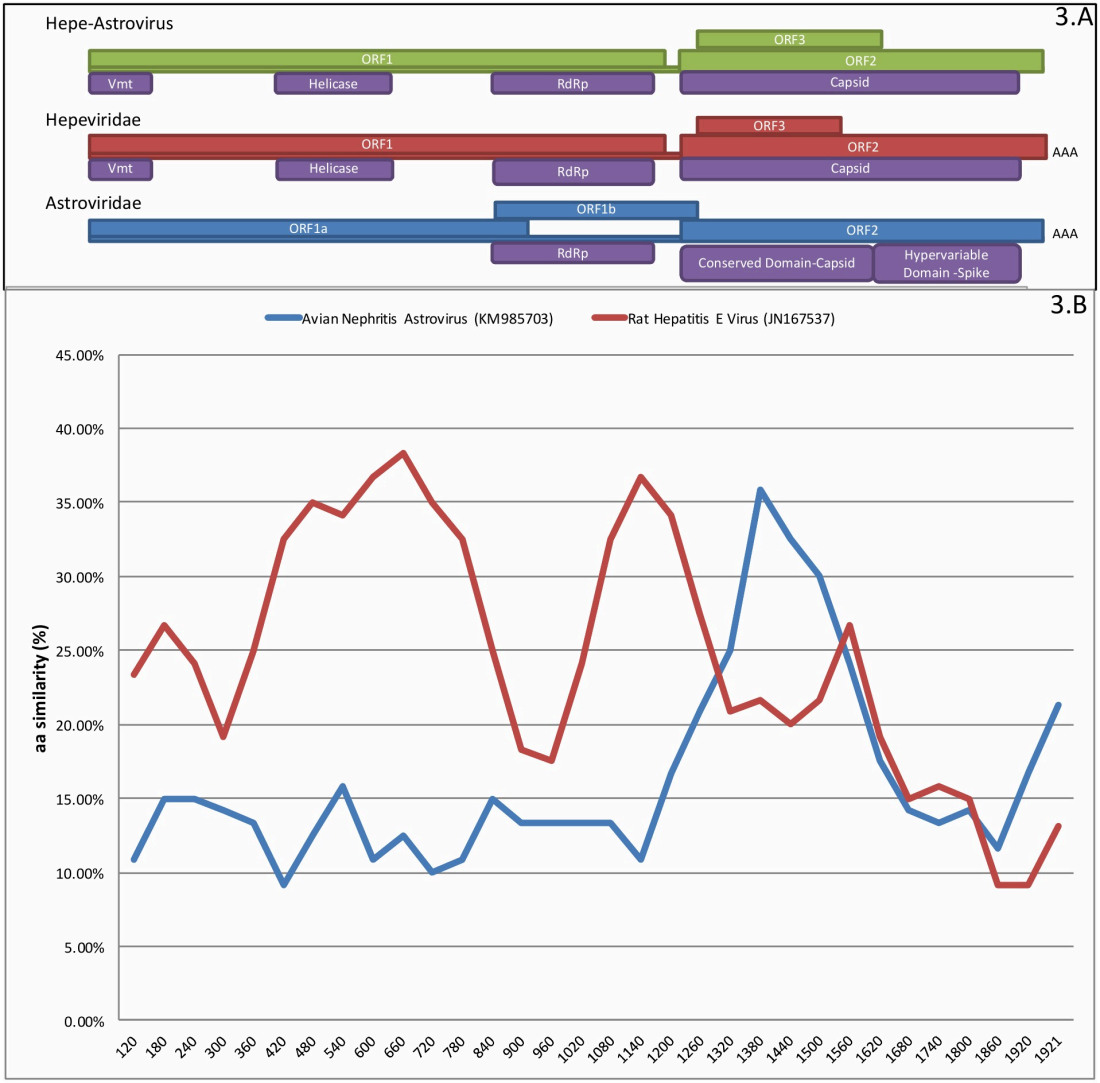
Genomic organization of a Middle East Hepe-Astrovirus. A. Genomic organization of ME Hepe-Astrovirus KSA 239, a related hepevirus (rat hepatitis E virus acc. no. JN167537) and an astrovirus (avian nephritis virus acc. no. KM985703) based on amino acid sequence. B. Simplot analysis using the Recombination Analysis Tool (RAT), illustrating amino acid homology between aligned sequences in a sliding window of 120 amino acids with an increment size of 60. x- axis is aa genomic position ORF1 and ORF2 for KS-239 Hepe-Astrovirus; y-axis is percent aa homology with a hepevirus (red line) and an astrovirus (blue line).

**Fig 4:**
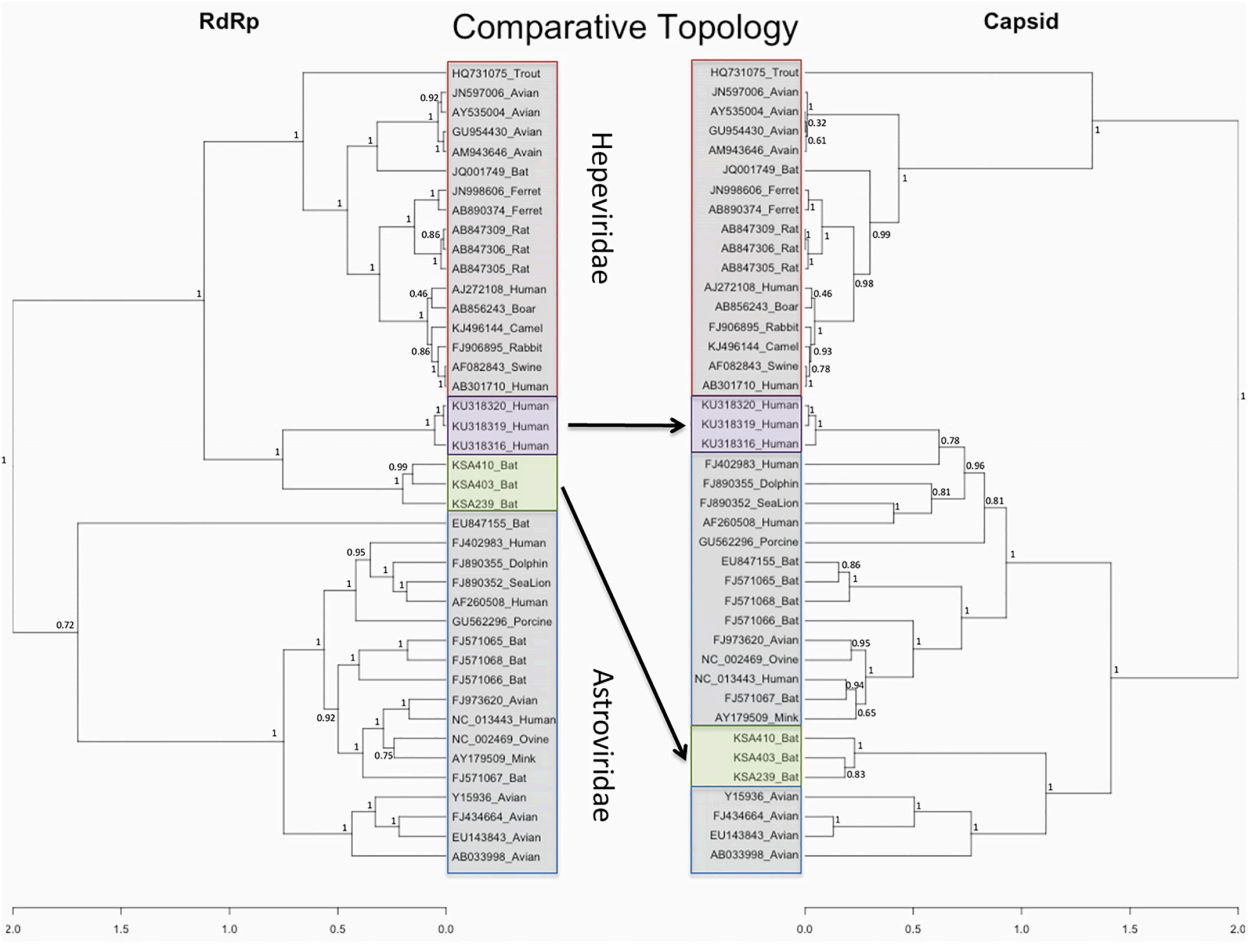
Comparative topology of ME Hepe-Astroviruses based on RdRp (left panel) and capsid protein (right panel). The data set was constructed using the KSA239, KSA 403 and KSA 410 ME hepe-astrovirus sequences along with 3 bastroviruses, 36 reference hepevirus and astrovirus genomes from Hepeviridae and Astroviridae viral families. All sequences selected have both an ORF1 (RdRp) and ORF2 (capsid) sequence available in NCBI database. Amino acids of both protein regions were aligned with MUltiple Sequence Comparison by Log-Expectation (MUSCLE), trimmed to 370 aa for RdRp and 240 aa for the capsid protein (conserved region). The phylogenetic trees were constructed using BEAST2 Bayesian phylogenetic software (13). Both analyses used the WAG substitution model (14), and were set with a strict molecular clock. The tangelogram (15) was constructed in R using the dendextend package and nodes are labeled with the posterior value (16).

We obtained two complete genomes and one nearly complete genome with homology to bastrovirus through a combination of HTS, 5’/3’ rapid amplification of cDNA ends (RACE PCR) and Sanger sequencing. Whereas KSA239 [complete genome, 5839 nt] was isolated from *R*. *hardwickii*, KSA403 [complete genome, 5745 nt] and KSA410 [partial genome, 4911 nt] were isolated from *E*. *helvum*. No poly-A tail was detected through HTS or 3’ RACE analysis. The genome organization of the three viruses is most similar to that of hepeviruses (Fig 3) rather than human bastrovirus. KSA239, KSA403, and KSA410 genomes showed ~45% nt homology and ~50% aa homology with human bastroviruses. Since the genomic organization of viruses recovered from KSA was more similar to hepeviruses and the nt/aa identity shared with bastroviruses was low, we describe these 3 viruses as ME Hepe-Astroviruses. ME Hepe-Astroviruses showed ~65% nt and ~70% aa homology with bat bastrovirus (acc. no. KX907131) and rat bastrovirus from Vietnam (acc. no. KX907127). They contain ORF1, ORF2 and ORF3. ORF1 and ORF2 are in frame; ORF3 is in +1 position and overlaps ORF2. They also contain conserved domains consistent with the viral methyltransferase (Vtm), helicase, RNA-dependent RNA polymerase (RdRp) and capsid proteins of hepeviruses. We also compared ORF1, ORF2 and ORF3 with other hepeviruses and astroviruses and found that whereas ORF1 had higher aa homology to *Hepeviridae* (30%-37%) than to *Astroviridae* (11%-19%), ORF2 had higher aa homology to *Astroviridae* (33%-36%) than to *Hepeviridae* (17%-21%) (Fig 3). KSA239, KSA403 and KSA410 showed 59.3%-63.7% nt identity with each other and had an average 61% aa homology, with highest homology in ORF1 (65%-70%) and lowest homology in ORF3 (57%-59%). Comparative topological analysis confirmed these findings. Whereas RdRp sequences clustered with *Hepeviridae*, the capsid proteins clustered with *Astroviridae* (Fig 4). Phylogenetic analysis for Hepe-Astroviruses identified a close lineage with human bastroviruses when comparing RdRp (ORF1) sequences, but a distant lineage with human bastroviruses when comparing capsid (ORF2) sequences (Fig 4).

#### Picornavirales

Approximately 50% of ssRNA viral reads were assigned to the order *Picornavirales* (Fig 2). We assembled all reads matching with *Picornavirales* with taxonomic classification (S2 Fig, http://krona.sourceforge.net) [35]. Approximately 60% of *Picornavirales* reads represented the family *Dicistroviridae* (invertebrate-borne picornaviruses that include black queen cell virus, himetobi P virus, and triatoma virus); ~13% represented the *Iflaviridae* (insect-borne picornaviruses); ~27% represented *Picornaviridae*.

#### A) Hepatoviruses

**Fig 5:**
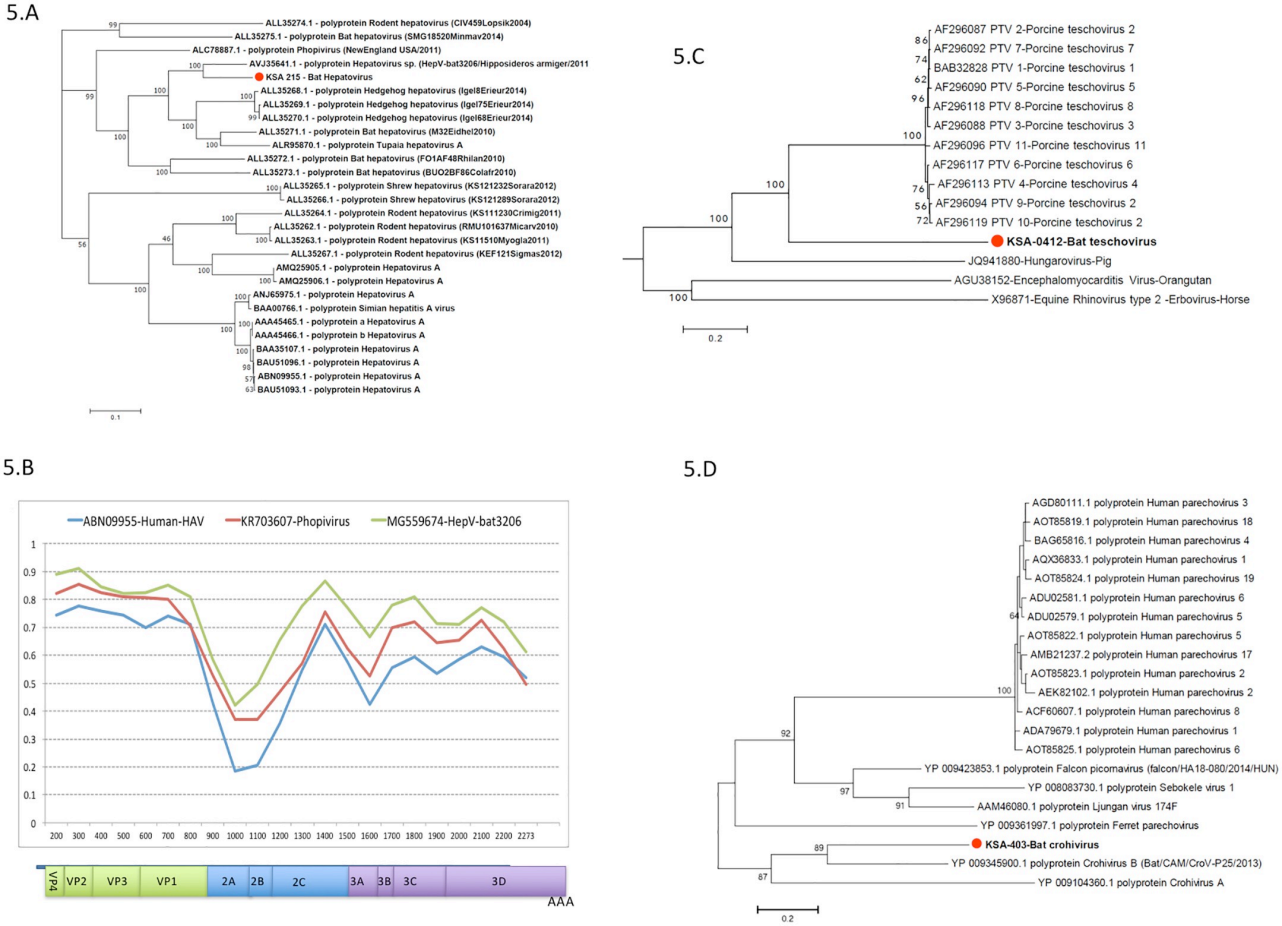
Phylogenetic analysis of hepatovirus KSA215, teschovirus KSA412, and Crohivirus KSA403, and recombination analysis of hepatovirus KSA 215. (A) Phylogenetic analysis of KSA215 bat hepatovirus based on amino acid sequences of complete polyprotein. (B) Simplot analysis using the Recombination Analysis Tool (RAT), illustrating amino acid homology between aligned sequences in a sliding window of 100 amino acids with an increment size of 100. x- axis is the polyprotein of KSA-215 Hepatovirus; y-axis is percent aa homology with a Human HAV (acc. no. ABN09955—blue line), bat HAV (acc. no. MG559674—green line) and phopivirus (acc. no. KR703607 -red line). (C) Phylogenetic analysis of KSA0412 bat teschovirus based on 971 amino acid long sequence of partial polyprotein (1246–2205 aa on polyprotein of PTV11, acc. no. AF296096). (D) The phylogenetic analysis of KSA0403 bat parechovirus based on 360 amino acid long sequence of partial polyprotein (1429–1788 aa on polyprotein of bat crohivirus, acc. no. YP009345900). All phylogenetic trees were created in MEGA7 software using maximum likelihood with 500 bootstrap replicates.

Six of 29 fecal samples from *R*. *hardwickii* had viral reads homologous with genus *Hepatovirus*. Based on viral reads obtained, we designed two consensus PCR assays targeting a partial region of 5’ NCR (356 nt) and VP2 (261 nt). 5’ NCR clones showed 99.3% nt identity and VP2 clones showed 98% nt and 100% aa identity among 6 positive samples (accession # MH367852-MH367863). We sequenced the complete genome of the PCR positive sample with the highest product signal (KSA-215, Accession # KX420952) using a combination of HTS data, 5’/3’ RACE and gap-filling PCRs. The KSA-215 hepatovirus genome was 7473 nt long with a single ORF encoding a 2229 aa long polyprotein. The genomic organization of KSA-215 hepatovirus was similar to those of other reported hepatoviruses [36, 37] (Fig 5) and consisted of a 5’ NCR, a polyprotein (VP4-VP2-VP3-VP1-2A-2B-2C-3A-3B-3C-3D proteins) and 3’ NCR with a poly ‘A’ tail (Fig 5). The KSA-215 hepatovirus showed 77% nt identity and 82.5% aa homology with a recently discovered hepatovirus identified in *Hipposideros armiger* bats from China [36] (Fig 5). The most similar human hepatovirus (acc. no. AAA45465.1) showed 63% nt and 74% aa identity with the KSA-215 hepatovirus. Phylogenetic analysis using complete polyprotein sequence of KSA-215 bat hepatovirus and other hepatoviruses revealed that the KSA-215 bat hepatovirus clusters with other bat and hedgehog hepatoviruses (Fig 5). Human and simian origin hepatoviruses formed a separate clade. Polyprotein sequences from KSA-215, bat hepatovirus (Bat-HepV, acc. no. MG559674), the closest human hepatovirus (acc. no. LC035020) and phopivirus (acc. no. KR703607) were analyzed using the recombination RNAlysis tool (RAT) (Fig 5). This analysis revealed that the KSA-215 bat hepatovirus was most similar to bat-hepatovirus through the polyprotein and had no evidence of recombination (Fig 5).

#### B) Teschoviruses

Suids (domestic pigs and wild relatives) are the only known natural hosts of teschoviruses. In four *E*. *helvum* fecal samples, viral reads revealed aa homology to porcine teschovirus. KSA-412 has a 3167 nt contig with a 971 aa sequence in the putative polyprotein that has only 47% aa homology to other known teschoviruses. When placed in the phylogenetic context of the picornavirus family (Fig 5), KSA-412 teschovirus clusters with the teschoviruses and hungaroviruses; encephalomyocarditis and erboviruses remaining as outgroups. This virus likely represents a new genus within the *Picornaviridae*.

#### C) Parechoviruses

**Fig 6:**
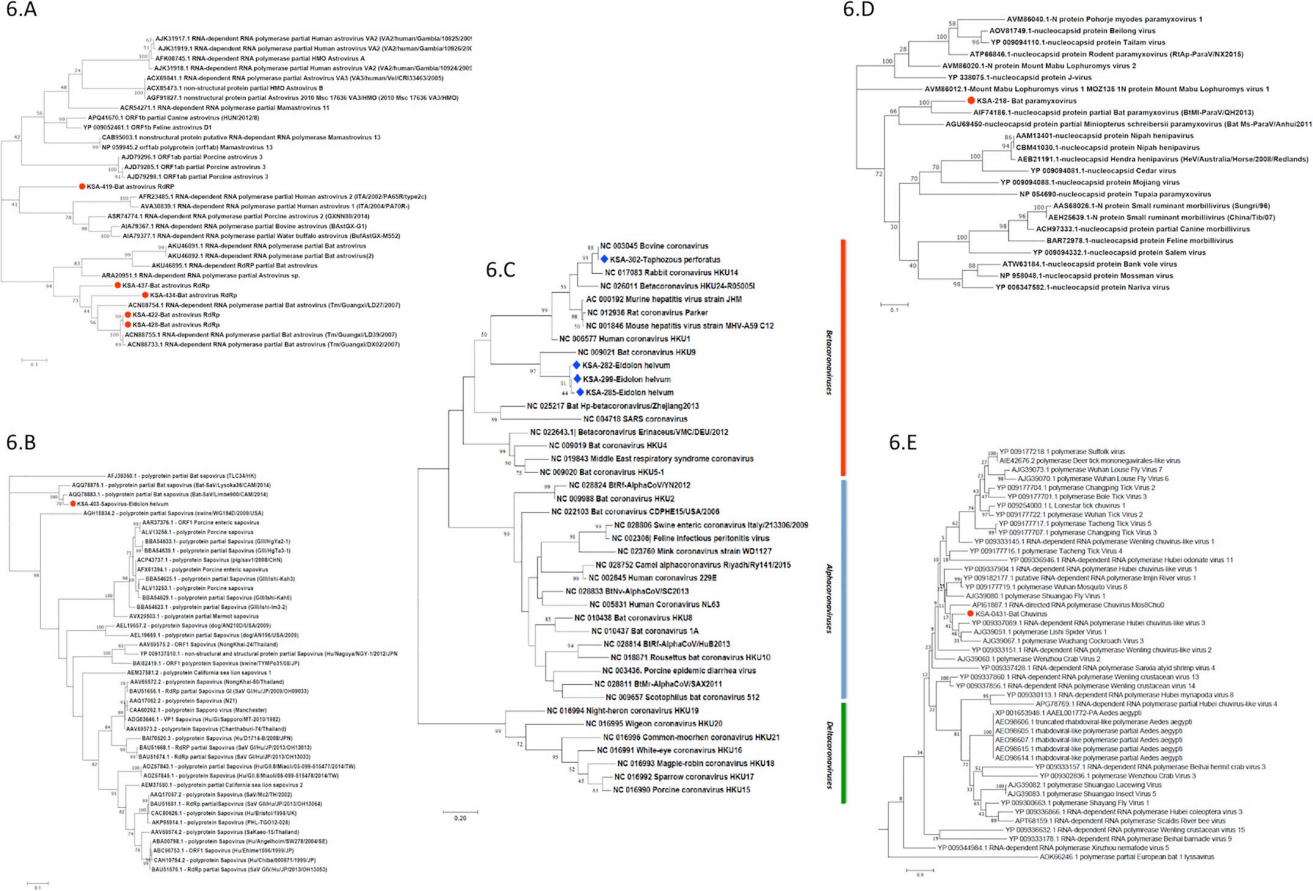
Phylogenetic analyses of astroviruses (KSA-419, KSA-428, KSA-422, KSA-434 and KSA-437), sapovirus KSA-403, and coronaviruses (KSA-302, KSA-282, KSA-299 and KSA-285), paramyxovirus KSA-218, and chuvirus KSA-431. (A) Phylogenetic analysis of KSA astroviruses based on 140 aa long partial RdRp region (268–407 aa on RdRp of Guang Xi bat astrovirus, acc. no. ACN88755); (B) Phylogenetic analysis of KSA Sapovirus based on 299 aa long partial RdRp region (1383–1680 aa on polyprotein of Cameroonian bat sapovirus, acc. no. AQQ78883); (C) Phylogenetic analysis of KSA Coronaviruses based on 139 aa long partial RdRp region (15177–15582 nt on ORF1ab of bovine coronavirus, acc. no. NC003045); (D) Phylogenetic analysis of KSA paramyxovirus based on 240 aa long partial nucleocapsid region (77–317 aa on nucleocapsid of bat paramyxovirus, acc. no. AIF74186); (E) Phylogenetic analysis of KSA Chuvirus based on 251 aa long partial RdRp region (685–936 aa on RdRp of Lishi spider virus, acc. no. AJG39051). All phylogenetic trees were created in MEGA7 software using maximum likelihood with 500 bootstrap replicates.

Parechovirus is another genus in the family *Picornaviridae*. Approximately 10% of total *Picornavirales* reads were similar to parechoviruses (21–86% at the nt level, 30–90% at the aa level), and 11% were most similar to Ljungan viruses (26–88% at the nt level, 31–95% at the aa, S3 Fig-Krona). We did not recover complete viral genomic sequence from any sample; however, a 1080 nt parechovirus contig was recovered from *E*. *helvum* (KSA403). This fragment encoded 360 aa of polyprotein corresponding to the 3A and peptidase C3 proteins. The KSA-403 parechovirus had 52% aa homology with a bat parechovirus (crohivirus) and 40% aa homology with a ferret parechovirus. The pairwise phylogenetic analysis of the KSA-403 parechovirus partial polyprotein with the corresponding protein regions of other parechoviruses is shown in phylogenetic Fig 6. Based on criteria established by the Picornavirus Study Group of the International Committee for Taxonomy of Viruses whereby members of the same species of the Parechovirus genus share > 70% aa identity in the polyprotein region, KSA-403 parechovirus appears to represent a new species in the *Parechovirus* genus, *Picornaviridae* family (Fig 5).

#### Astroviridae

Four *T*. *perforatus* samples and one *E*. *helvum* sample contained astrovirus sequences. We amplified a portion of the RdRp region using PCR [38]. Sequences of the resulting ~420 nt RdRp products corresponding to 140 aa have been submitted to Genbank (acc. no. MH424428-MH424433). A sequence from *E*. *helvum* (KSA-419) was most closely related (70% aa homology) to a bovine astrovirus. Astroviruses from *T*. *perforatus* (found in KSA-422, KSA-428, KSA-434, and KSA-435) had >95% aa homology across the entire genome to an astrovirus discovered in a *T*. *melanopogon* from China in 2007 [39]. RdRp based protein phylogenetic analysis showed that astroviruses from KSA-422, KSA-428, KSA-434 and KSA-435 clustered with other bat astroviruses; the KSA-419 astrovirus clustered with other reported bovine, porcine and human astroviruses (Fig 6).

#### Sapoviruses

Five of 24 *E*. *helvum bats* contained reads that matched viruses from the genus *Sapovirus*. One fecal sample yielded an 899 nt contig (KSA-403 bat sapovirus) in the RdRp with 82% nt and 99% aa homology to a partial sapovirus sequence retrieved from a *E*. *helvum* bat in Cameroon (acc. no. AQQ78883) [40]. Phylogenetic analysis demonstrated a closer relationship between KSA-403 and other bat sapoviruses than with human or porcine sapoviruses (Fig 6).

#### Coronaviruses

Four bat fecal samples (three *E*. *helvum* and one *T*. *perforatus*) revealed the presence of betacoronavirus sequences. Contig lengths derived from HTS data were insufficient for phylogenetic analyses, thus we amplified ~400 nt RdRp coding fragments by consensus PCR [41]. Sequence from KSA302 (400 nt), recovered from a *T*. *perforatus* bat, had 100% nt identity with a bovine coronavirus from Louisiana, USA (acc. no. NC003045). Three additional 400 nt products from *E*. *helvum* bats had 99–100% nt homology with a *Rousettus* bat betacoronavirus HKU9 from China (acc. no, NC009021) (Fig 6).

### Negative sense RNA viruses

One *R*. *hardwickii* fecal sample yielded paramyxovirus reads (KSA-218). We recovered a 713 nt fragment of nucleocapsid protein that had 72% nt and 75% aa homology with bat paramyxovirus isolate BtMl-ParaV/QH2013 from China (acc. no. AIF74186) (Fig 6). One *T*. *perforatus* bat had sequences consistent with a novel chuvirus (KSA-431). Analysis of a 762 nt RdRp fragment revealed 65% nt identity with Lishi Spider Virus 1 (acc. no. KM817597) and 52% aa homology with the RdRp of Hubei chuvirus-like virus 3 (acc. no. YP_009337089). KSA-431-Chuvirus clustered with other arthropod-borne negative sense RNA viruses in phylogenetic analysis (Fig 6).

### Double stranded RNA viruses

#### Bat Rotavirus

Approximately 13% of viral reads represented dsRNA viruses (Fig 1). Twenty-seven percent of dsRNA reads were similar to sequences from family *Reoviridae*; the remainder matched most closely with plant, bacterial, fungal or insect dsRNA viruses including drosophila A virus, circulifer tenellus virus 1, spissistilus festinus virus, partitivirus, totivirus, unclassified partitiviridae, unclassified birnaviridae, lentinula edodes helical virus and picobirnavirus (Fig 2). Approximately 2% of dsRNA sequences had nt similarity with rotaviruses. Five fecal samples from *E*. *helvum* (n = 3) and *R*. *hardwicki* and *T*. *perforatus* (each n = 1) had rotavirus A (RVA) reads. Based on these reads, we designed a consensus VP1 PCR assay to test for presence of bat RVA sequences. We detected 5 VP1 amplification products that had >99% nt identity with one another and 96–97% nt and 98% aa homology with a recently discovered Rotavirus A sequence (Bat-wt/CMR/BatLy03/2014/G25P43, acc. no. KX268776) from *E*. *helvum* bats in Cameroon [10]. We recovered a complete RVA genome from an *E*. *helvum* sample (KSA-402) using HTS data, 5’/3’ RACE and gap filling PCR. The KSA-402 RVA genome consisted of 11 dsRNA fragments ranging in size from 528 bp to 3267 bp (S1 Table). Stoichiometric analyses based on Sybrgreen realtime assays for each of the 11 individual RNA segments indicated that the number of RNA copies per reaction was similar for each segment (S3 Fig and S2 Table). These findings are consistent with the presence of a single reovirus [42]. VP7 segment of KSA-402 RVA showed 83% nt homology with the VP7 segment of Bat-wt/CMR/BatLy03/2014/G25P43. The remaining 10 segments showed 97–100% nt homology with Bat-wt/CMR/BatLy03/2014/G25P43 (acc. no. KX268777-KX268786). Whereas KSA-402 RVA is also similar to other bat rotaviruses (bat/4852/Kenya/2007 for the VP2, VP6, VP7, NSP2, NSP3, and NSP5 segments), the VP1, VP3, VP4, NSP1, NSP4 segments are similar to a diverse array of mammalian RVAs: VP1 had 79% aa similarity with a human rotavirus (genotype G10P/11) from India; VP3 had 74% aa identity with a Nigerian-origin cow rotavirus (genotype G8P); VP4 had 76% aa homology with a Nigerian-origin human rotavirus (genotype G8P); NSP4 had 74% aa level homology with a human rotavirus (genotype B10); and NSP1 had 36% aa homology with human rotavirus (genotype B10) (S1 Table). Five of eleven KSA-402 rotavirus segments had less than 80% aa homology to known human rotaviruses; our findings are compatible with the discovery of a new rotavirus genotype [43].

### DNA viruses

Twenty-seven percent of 0.24 million viral reads represented DNA viruses (ssDNA viruses: ~5.6 million reads, 23.2% and dsDNA viruses: ~1 million reads, 4.1%) (Fig 1). Approximately 45% of ssDNA virus reads matched with insect-borne densoviruses, ~27% ssDNA reads matched with unclassified circoviruses and 25% reads matched with unclassified parvoviruses. We did not perform further analyses of these diverse viral taxas of ssDNA viruses. In dsDNA virus most reads matched with family *Adenoviridae* (~41%), *Polyomaviridae* (~15%), *Papillomaviridae* (~10%) and unclassified papillomaviruses (~14%) (Fig 2).

#### Polyomaviruses

**Fig 7:**
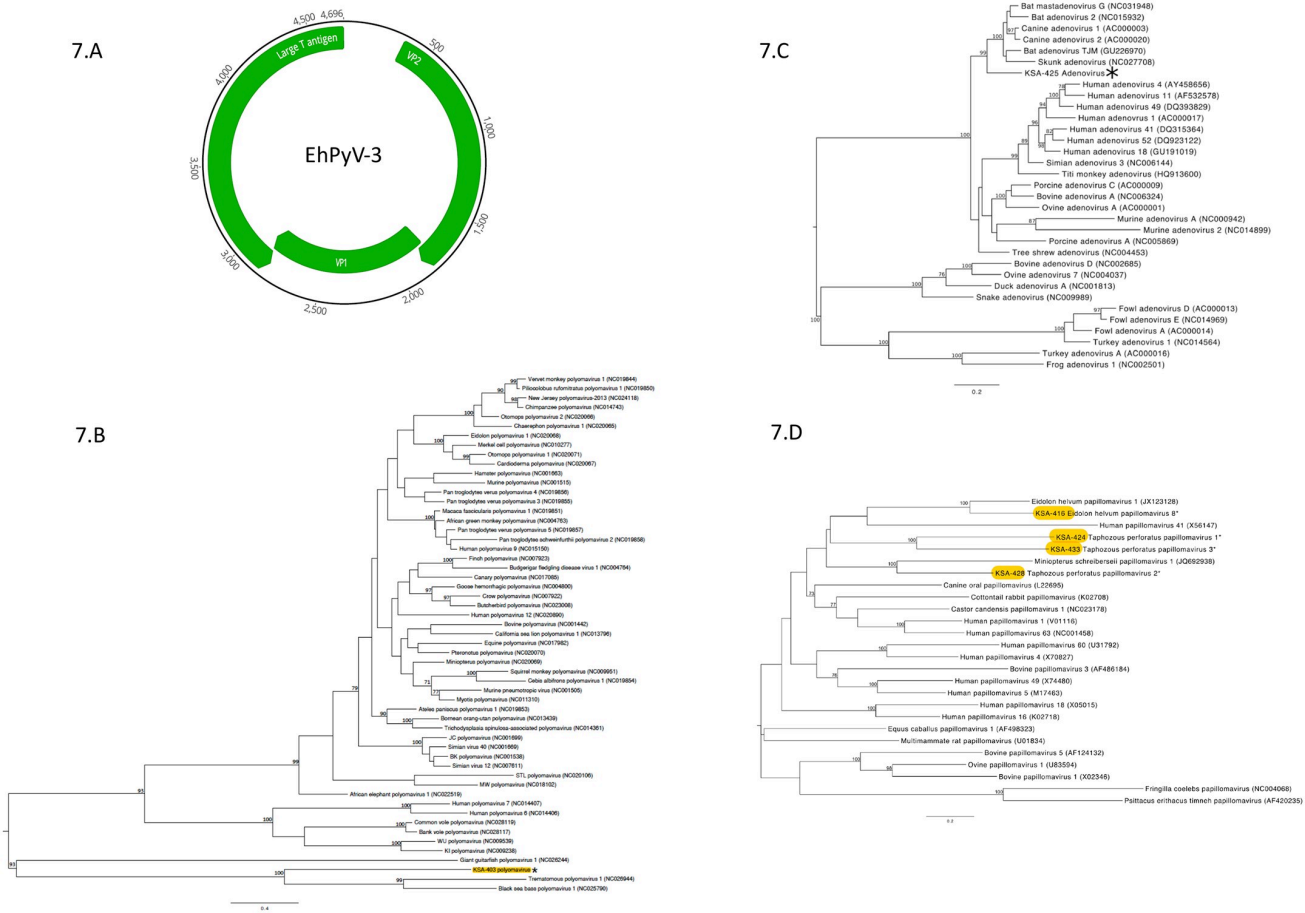
Genomic organization of polyomavirus and phylogenetic analysis of dsDNA viruses. (A) Genome structure of KSA403 (EhPyV-3). Numbers represent nucleotide positions; green arrows indicate coding regions for each gene. Maximum likelihood phylogenetic tree of the (B) VP1 protein for representative members of the family Polyomaviridae (KSA-403) (C) Hexon protein for representative members of the family Adenoviridae with KSA-425 (D) VP1 protein for representative members of the family Papillomaviridae (KSA-416, KSA-424, KSA-433 and KSA-428). For all Maximum likelihood phylogenetic trees, the scale bar represents units of substitutions per site. An asterisk denotes the virus discovered in this study. Bootstrap nodal support values are indicated if > 70.

A novel polyomavirus was identified in *E*. *helvum* that represents the third polyomavirus identified in this bat species. The complete sequence of *E*. *helvum* polyomavirus 3 (KSA-403, EhPyV-3, acc. no. KX434762) was recovered from HTS data and confirmed using overlapping PCRs followed by bidirectional Sanger sequencing (Fig 7). The primers for complete genome sequencing of EHPyV-3 were designed using HTS data. EhPyV-3 is a circular dsDNA virus with a 4696 bp genome encoding three proteins; VP1, VP2 and large T antigen. EhPyV-3 is most similar to Pomona leaf-nosed bat (*Hipposideros pomona*) associated polyomavirus with 73% aa similarity for VP1, 66% for VP2 and 68% for the large T antigen. According to ICTV guidelines, EhPyV-3 meets criteria for classification as a new species [44]. Phylogenetic analysis of the VP1 protein places EhPyV-3 in a well-supported clade (99% bootstrap nodal support) with Pomona leaf-nosed bat associated polyomavirus (Fig 7). These two viruses do not cluster with any of the four existing polyomavirus genera; instead, they share a basal node with unassigned fish viruses.

#### Adenoviruses

The partial genomic sequence of an adenovirus was identified in a *T*. *perforatus* bat (KSA-425, acc. no. KX434765) that included the complete coding sequence (2700nt) for the 899 aa long hexon protein. The complete hexon gene found in sample KSA-425 was confirmed using overlapping PCRs and bidirectional Sanger sequencing. The primers for complete hexon sequencing were designed using HTS data. *T*. *perforatus* adenovirus 1 is the first adenovirus to be identified in this bat species and shares 81% aa similarity across the hexon protein with canine adenovirus 1 and bat mastadenovirus G (acc. number NC031948). Bat mastadenovirus was identified in Rafinesque’s big-eared bat (*Corynorhinus rafinesquii*) in the United States [45]. Phylogenetic analysis of the hexon protein revealed that *T*. *perforatus* adenovirus 1 clusters with other bat mastadenoviruses, as well as skunk and canine adenoviruses (Fig 7). The *T*. *perforatus* adenovirus 1 hexon protein is the shortest amongst other members of this clade.

#### Papillomaviruses

Four distinct papillomavirus sequences were identified in two bat species, *E*. *helvum* (*Eidolon helvum papillomavirus* 8; EhPv-8) and *T*. *perforatus* (*T*. *perforatus* papillomavirus 1, 2 and 3; TpPv-1, -2, -3). Complete coding sequences were recovered for EhPv-8 (acc. no. KX434763, 6985 bp), TpPv-1 (acc. no. KX434764, 7180 bp) and TpPv-3 (acc. no. KX434767, 7083bp). EpPv-8 shared 63% aa similarity to *E*. *helvum* papillomavirus type 1 across the DNA helicase protein (E1). TpPv-1, TpPv-2, and TpPv-3 (acc. no. KX434766, partial genome) shared 44%, 47% and 44% aa similarity across the E1 protein with human papillomavirus 63, *Miniopterus schrebersii* papillomavirus 1, and *Castor canadensis* papillomavirus 1, respectively (Fig 7).

## Discussion

Nearly three quarters of newly emerging and re-emerging zoonoses are linked to wildlife. Bats harbor a greater proportion of zoonotic viruses per host species than other mammalian orders [5]. In some instances outbreaks have been linked to bat roosting or foraging in close proximity to human settlements [7, 11, 46, 47]. The 2002–2003 SARS CoV outbreak in China, which caused more than 8000 cases of severe respiratory disease in humans resulting in ~ 800 fatalities and profound economic losses [48, 49], was linked to *Rhinolophus* sp. bats and the wildlife trade [50]. In 2012, the KSA reported a case of fatal pneumonia associated with a novel coronavirus, MERS-CoV infection [51]. MERS-CoV continues to cause morbidity and mortality chiefly in the Middle East and in travelers returning from the Middle East [51]. While primary human cases of MERS-CoV are often associated with epidemiological exposure to infected dromedary camels [52, 53], phylogenetic analyses and the global distribution of MERS-CoV related viruses in bats suggests that they were likely an original source of spillover to camels [54, 55].

Our current metagenomic study increases the knowledge of bat viruses circulating in the KSA. Only ~0.52% of total Illumina reads matched with viral reads and the majority of viral reads in bats samples likely reflect their diets. Approximately 83% of total viral sequences were related to phages, insect borne viruses and plant viruses, and only 14% represented mammalian viruses.

The Hepe-Astroviruses found in *E*. *helvum* and *R*. *hardwickii* likely represent members of a new genus in the family *Hepeviridae* based upon sequence and genomic organization. The ME Hepe-Astrovirus from *R*. *hardwickii* (KSA 239) showed 63–65% aa homology with Hepe-Astroviruses from *E*. *helvum* (KSA 403, and KSA 410). *E*. *helvum* and *R*. *hardwickii* are phylogenetically and ecologically distinct bat species: *E*. *helvum* is a large widely roaming frugivorous bat that roost on trees while *R*. *hardwickii* is an insectivorous cave-roosting bat. These viruses have ~42–53% nt sequence identity to unpublished Bastroviruses from rats, pigs and bats from Vietnam, and the Bastro-like virus recently discovered in *E*. *helvum* bats that has been described as a novel astrovirus [9]. Whereas the ME Hepe-Astrovirus ORF1 is most homologous to hepeviruses, its ORF2 is most homologous to astroviruses (Fig 3B). Astroviruses have three overlapping ORFs; ORF1a, ORF1b and ORF2. In contrast, ME Hepe-Astroviruses have a genomic organization more similar to *Hepeviridae*: the ORF1 and ORF2 are non-overlapping but ORF3 is overlapping with ORF2 (Fig 3A). All three ME Hepe-Astroviruses identified in this study have a 10-nt long non-coding region between ORF1 and ORF2. The ME Hepe-Astrovirus ORF3 is characteristic of the *Hepeviridae* family, but not of the *Astroviridae* family [56]. Dryden et al. proposed a phylogenetic relationship between the *Astroviridae* and *Hepeviridae* viral families based on the structural similarities between immature human Astrovirus and hepatitis E like particles [57]. The ORF2 capsid protein of ME Hepe-Astroviruses shares very low aa identity with the hepeviruses, while the ORF1 protein showed very low aa similarity to astroviruses. The ORF1 non-structural protein in ME Hepe-Astroviruses contains functional domains of Hepeviridae and the phylogenetic and genomic analysis of the ME Hepe-Astroviruses consist with recombination events for previously reported bastroviruses [9, 34]. Thus, like bastroviruses, ME Hepe-Astrovirus sequences also support the argument for a common ancestor between the *Astroviridae* and *Hepeviridae* families. We also discovered five bat astroviruses. The majority of astroviruses found in *T*. *perforatus* bats from KSA are homologous to bat astroviruses discovered in *T*. *perforatus* bats from China. However, we also found an astrovirus in *E*. *helvum* [KSA-419] that was more closely related to bovine astroviruses.

The genomic organization of KSA-215 hepatovirus was similar to those of other reported hepatoviruses [36, 37] (Fig 5A). The KSA-215 hepatovirus showed 77% nt identity and 82.5% aa homology with a recently discovered *Hipposideros armiger* bat hepatovirus from China, (Fig 5A). The closest human hepatovirus (acc. no. AAA45465.1) shared 63% nt and 74% aa identity with the KSA-215 hepatovirus. *R*. *hardwickii* and *H*. *armiger* bat species have no geographical range overlap and are from two divergent families, however homology between hepatoviruses found in these two species indicates a recent common ancestor or a wider viral host range that involves intermediate bridge hosts. Suids (domestic and wild pigs) are the only known natural hosts of teschoviruses [58]. However, we identified viral reads homologous to porcine teschoviruses in four *E*. *helvum* bats (KSA-412). We speculate that the bat teschovirus detected in KSA-412 will ultimately be described as representing a new genus within the *Picornaviridae*. Based on criteria established by the Picornavirus Study Group of the International Committee for Taxonomy of Viruses, KSA-403 parechovirus (bat crohivirus) also appears to represent a new species in the *Picornaviridae* family. Three betacoronaviruses from *E*. *helvum* bats were more than 98% homologous at the nt level with a *R*. *aegyptiacus* bat betacoronavirus 2d from China (bat coronavirus HKU9-1, acc. no, NC009021). One *T*. *perforatus bat* (KSA-302) coronavirus had 100% nt homology with a bovine coronavirus from Louisiana, USA (acc. no. NC003045), that was isolated from an animal experiencing fatal pneumonia during a bovine shipping fever epizootic.

Reads and contigs that matched with rotaviruses (RV) and unclassified picobirnaviruses were the only dsRNA vertebrate viruses found in this study. These included RVs detected in *R*. *hardwickii*, *T*. *perforatus*, and *E*. *helvum* bats. Similar RVs were recently discovered in Cameroonian *E*. *helvum* bats (Bat-wt/CMR/BatLy03/2014/G25P43). Ten out of 11 segments from KSA-402 RVA showed >97% nt and >98% aa homology with the Cameroonian *E*. *helvum* RVA. VP7 had 83% nt and 86% aa homology with Cameroonian *E*. *helvum*. Bisha, KSA and Cameroon are separated by 4500 km. The presence of similar RVAs across the geographic range of *E*. *helvum*, known to fly long distances [59], suggest spread and introduction of these viruses during migration and flight—even to the most geographically isolated portion of its range in KSA.

The next nearest known host to *E*. *helvum* polyomavirus 3 is the insectivorous Pomona leaf-nosed bats (*Hipposideros pomona*), a species found in the northern regions of South East Asia as well as parts of southern China, Nepal and Bangladesh [60]. These bat species have no geographical overlap, as *E*. *helvum* is restricted to Africa, Yemen and Saudi Arabia. These data suggest an underappreciated diversity of polyomaviruses remains to be discovered in bats sampled from intermediate geographical locations, including wide swaths of Western Asia that remain relatively unexplored. The discovery of near complete genomes for four papillomaviruses, including three from *T*. *perforatus*, demonstrates co-circulation of multiple genotypes in a localized region of KSA, and expands the collection of the highly diverse *Papillomaviridae* viral family. We also detected the first adenovirus of *T*. *perforatus* that was related to canine adenovirus, and a bat adenovirus found in USA.

In summary, as in other geographic catchment areas, bats endemic in the KSA have richly diverse viromes. The most remarkable single finding was the presence of Hepe-Astroviruses, that are similar to Bastroviruses and bastro-like viruses but with a distinctive hepevirus-like genomic organization. Further molecular and serological studies are required to test whether these viruses are capable or infecting humans, domestic animals, or other wildlife.

## Conclusions

Although bats provide invaluable ecosystem services including insect control, pollination, and seed dispersal, they harbor a higher proportion of zoonotic viruses as compared to other mammalian orders. The majority of current bat virome projects are focused on bat populations from temperate or tropical regions of the world, particularly in Asia, North America, and Europe. This is the first bat virome study from the Arabian Peninsula and comprises four bat species endemic in the KSA. Using high throughput sequencing methods, we found a diverse flora of viruses in these bats, including a bastro-like virus (Middle East Hepe-Astrovirus) that suggests a common ancestor for astroviruses and hepeviruses.

## Supporting information

S1 Table Genome segments analysis of KSA-402 Rotavirus A. (PDF)

S2 Table Primers used for SYBR green PCR for KSA-402 Rotavirus (segments 1–11). (PDF)

S1 Fig Comparative abundance of sequences representing viral taxa within the larger categories of single stranded RNA viruses (A), Single stranded DNA viruses (B), Double stranded DNA viruses (C), and Double stranded RNA viruses (D) for the four bat species *E*. *helvum*, *R*. *hardwickii*, *R*. *aegyptiacus*, *and T*. *perforatus*. (PDF)

S2 Fig Distribution of reads within the order Picornavirales based on KRONA analysis (Ondov, Bergman, & Phillippy, 2011). The majority of reads were distributed within the families Dicistroviridae (red) and Iflaviridae (dark green). Picornaviridae (light green) represented a wide range of vertebrate picornaviruses.
(PDF)

S3 Fig KSA-402 bat rotavirus SYBRgreen real-time PCR Ct values for all 11 segments; x-axis- notation of 11 rotavirus segments (VP1, VP2, VP3, VP4, VP6, VP7, NSP1, NSP2, NSP3, NSP4 and NSP5): y-axis- each blue dot on red line represent Ct value for each related segment on x-axis in the same KSA-402 RNA extract. (PDF)
