# Supplementary material for: A viral metagenomic survey identifies known and novel mammalian viruses in bats from Saudi Arabia
Source: PLoS One. 2019 Apr 10;14(4):e0214227. doi: 10.1371/journal.pone.0214227 (PMC6457491; doi:10.1371/journal.pone.0214227)
Supplement: S1 Table — (PDF) [file pone.0214227.s001.pdf]

Gene	KSA-402 rotavirus fragment (bp)	Related Rotavirus isolate for genotyping based on NCBI database	% Identity based on blastX	NCBI accession number	Genotype
VP1	3267	Rotavirus A strain RVA/Human-wt/IND/N284/2004/G10P[11]	79%	KC175028	NEW (R)
VP2	2712	Rotavirus A bat/4852/Kenya/2007	96%	GU983673.1	C8
VP3	2508	RVA/Cow-tc/NGA/NGRBg8/1998/G8P[1]	74%	LC119106.1	NEW (M)
VP4	2331	RVA/Human-tc/NGA/HMG035/1999/G8P[1]	76%	AF361438	NEW (P)
NSP1	1689	Human rotavirus A strain B10	36%	ADP68537.1	NEW (A)
VP6	1194	Rotavirus A bat/4852/Kenya/2007	97%	GU983675.1	I15
NSP2	954	Rotavirus A bat/4852/Kenya/2007	97%	GU983677.1	N8
VP7	981	Rotavirus A bat/4852/Kenya/2007	83%	GU983676.1	G25
NSP3	936	Rotavirus A bat/4852/Kenya/2007	96%	GU983678.1	T11
NSP5	630	Rotavirus A bat/4852/Kenya/2007	96%	GU983680.1	H10
NSP4	528	Human rotavirus A strain B10	74%	HM627562.1	NEW (E)
