# Supplementary material for: A viral metagenomic survey identifies known and novel mammalian viruses in bats from Saudi Arabia
Source: PLoS One. 2019 Apr 10;14(4):e0214227. doi: 10.1371/journal.pone.0214227 (PMC6457491; doi:10.1371/journal.pone.0214227)
Supplement: S2 Table — (PDF) [file pone.0214227.s002.pdf]

Segment Number	Segment Name	Accession Number	Primer Name	Primer Sequence	Position	Amplicon Length (bp)
1	VP1	KX420939	RV-VP1-F1	GTACGTGCAATAGTACCAGAC	896-916	244
			RV-VP1-R1	CGTCAACATCGTAAGTATACTCAG	1139-1116	
2	VP2	KX420940	RV-VP2-F1	GTTATGACGCAGAAAGAGGAAG	192-213	244
			RV-VP2-R1	TAGTCTGCTTCTTAGCCTGC	435-416	
3	VP3	KX420941	RV-VP3-F1	GAAAGATGGCTTGGACGAC	821-839	226
			RV-VP3-R1	GGTGTTTATTGGAGTACGGTG	1046-1026	
4	VP4	KX420942	RV-VP4-F1	CCACATGCTCAGTGAATGG	1049-1067	269
			RV-VP4-R1	ATGTAATGTTACGGCACCACC	1317-1297	
5	NSP1	KX420943	RV-NSP1-F1	CGCTGATTCGCCAATGTAC	1298-1316	253
			RV-NSP1-R1	GGAATATCTACCATTCAGCGAG	1550-1529	
6	VP6	KX420944	RV-VP6-F1	GTAATGGTATTGCGCCACAATC	748-769	206
			RV-VP6-R1	ATCATGCTGTGGCTGTGATC	953-934	
7	NSP2	KX420945	RV-NSP2-F1	GCAACAGCTGAAGGTGG	533-549	231
			RV-NSP2-R1	GTAATGACCCTTTCCATGCG	763-744	
8	VP7	KX420946	RV-VP7-F1	GACCAAGAACTCGATATGTCAG	632-653	234
			RV-VP7-R1	CAGTTTCTCCTGTGTTGCG	865-847	
9	NSP3	KX420947	RV-NSP3-F1	CAGTCAGTGTTGAACTCTGC	164-183	239
			RV-NSP3-R1	GAGTCAGTCATCCAATCCC	402-384	
10	NSP5/6	KX420948	RV-NSP56-F1	CGACAGCGTCAACTCTTTC	125-143	255
			RV-NSP56-R1	CTACTTGGTCGCATCCC	379-363	
11	NSP4	KX420949	RV-NSP4-F1	CCAACAGCAAAGATGGCTC	154-172	234
			RV-NSP4-R1	CCTCTTGAGCAGTTCGAC	387-370	
