# Supplementary figures and images for: A viral metagenomic survey identifies known and novel mammalian viruses in bats from Saudi Arabia
Source: PLoS One. 2019 Apr 10;14(4):e0214227. doi: 10.1371/journal.pone.0214227 (PMC6457491; doi:10.1371/journal.pone.0214227)

S1.

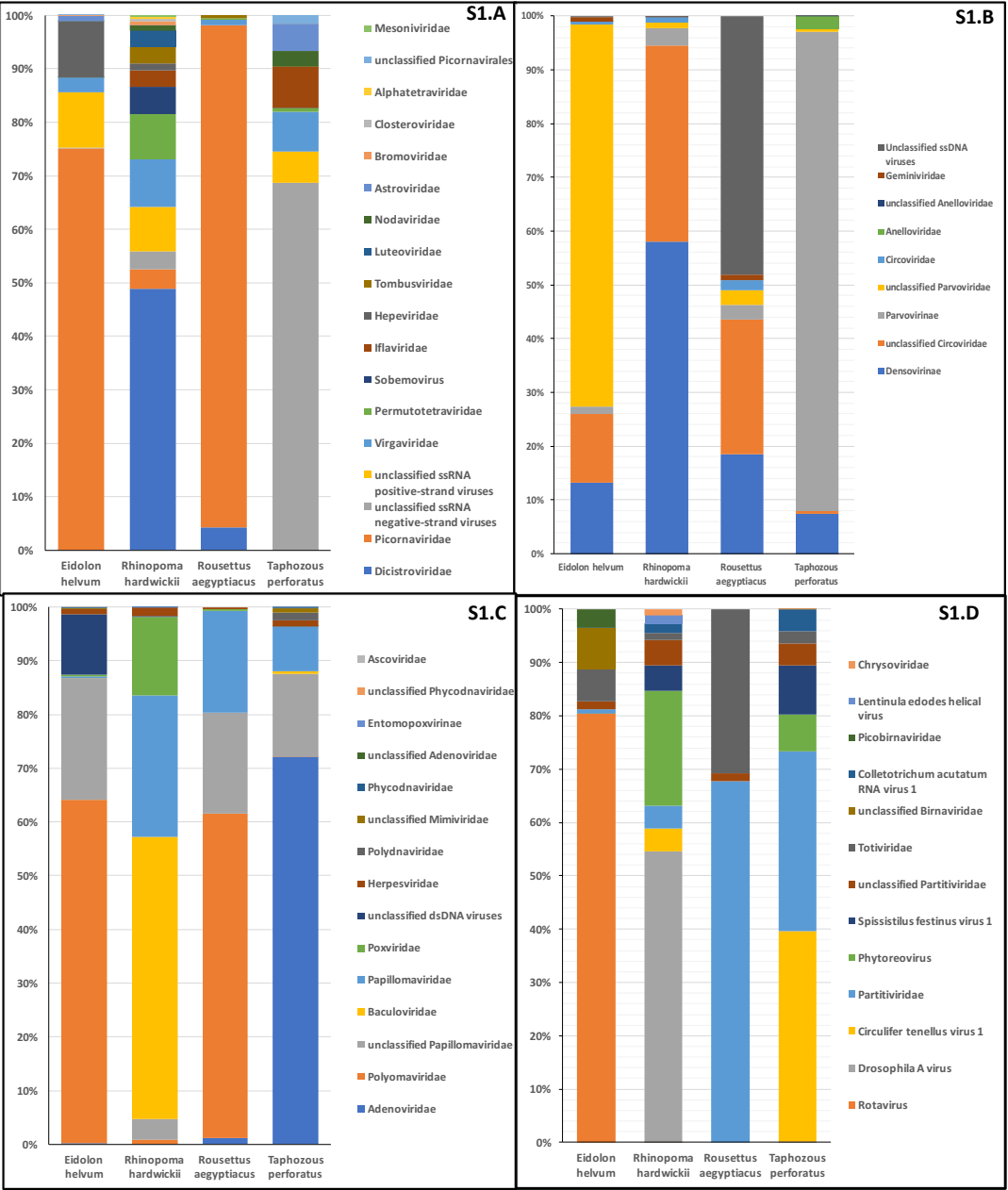

Supplement: S1 Fig — (PDF) [file pone.0214227.s003.pdf]

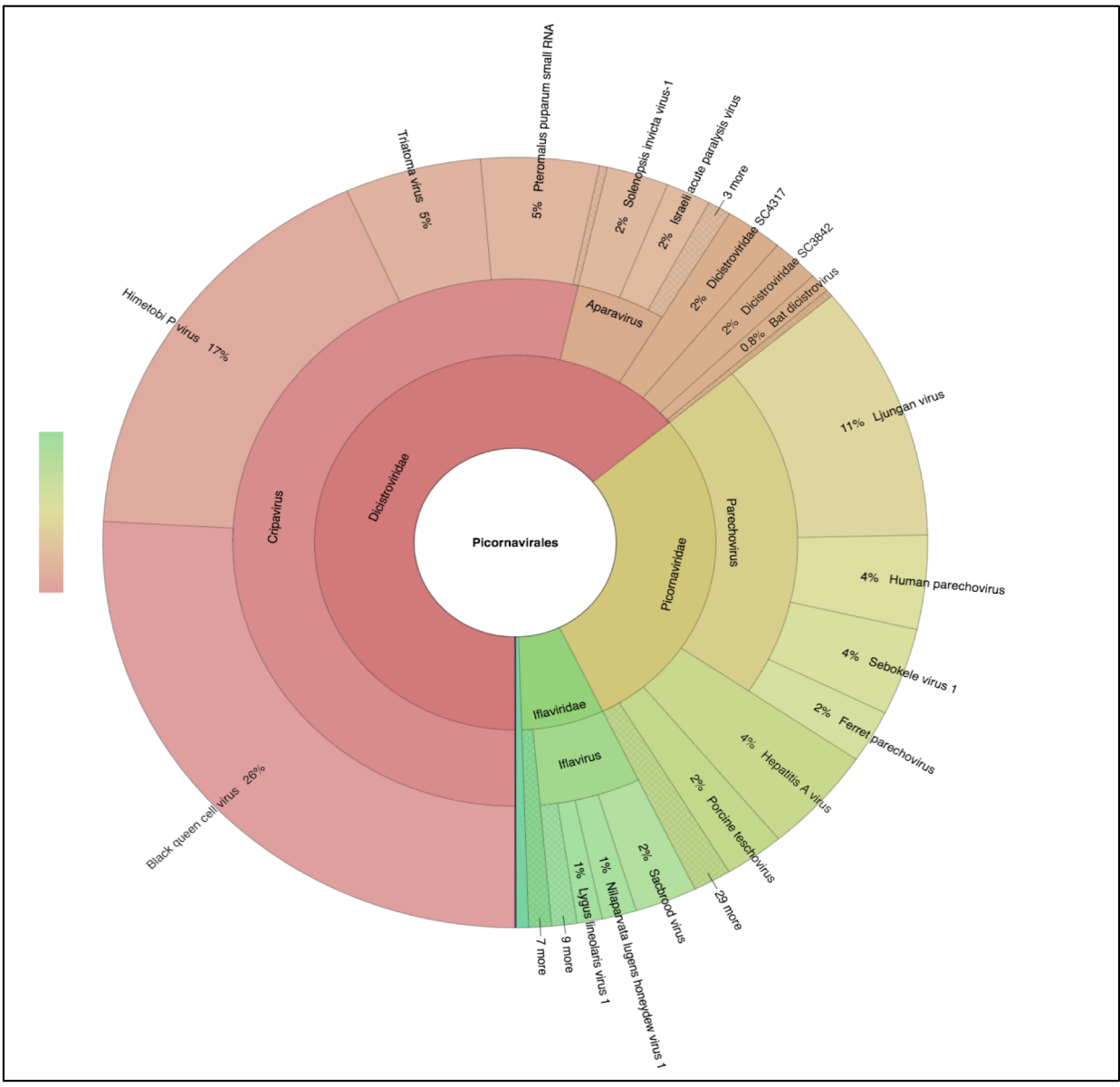

Supplement: S2 Fig — The majority of reads were distributed within the families Dicistroviridae (red) and Iflaviridae (dark green). Picornaviridae (light green) represented a wide range of vertebrate picornaviruses. (PDF) [file pone.0214227.s004.pdf]

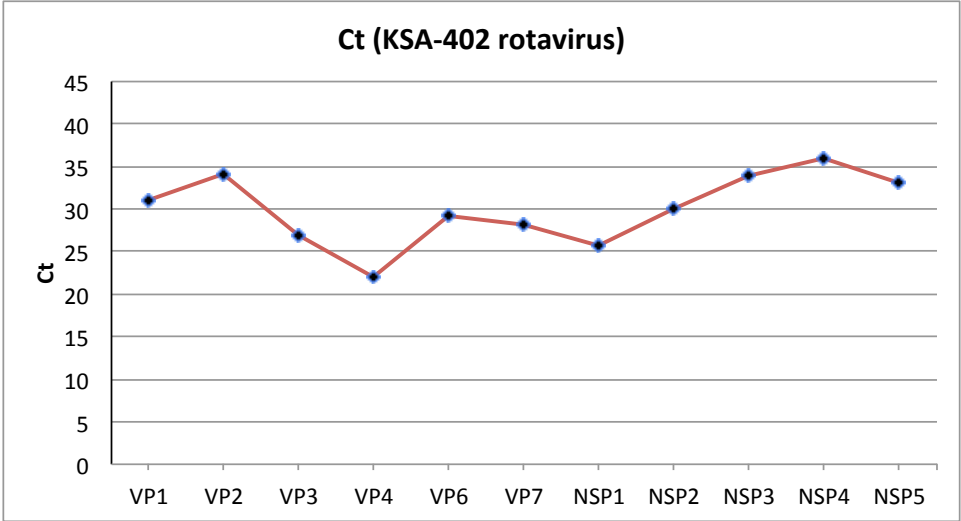

Supplement: S3 Fig — (PDF) [file pone.0214227.s005.pdf]
